# Detoxification of coumarins by rumen anaerobic fungi: insights into microbial degradation pathways and agricultural applications

**DOI:** 10.1186/s40104-025-01195-9

**Published:** 2025-04-17

**Authors:** Yuqi Li, Jian Gao, Yaxiong Cao, Xinming Cheng, Zhanying Sun, Jiyu Zhang, Weiyun Zhu, Martin Gierus, Yanfen Cheng

**Affiliations:** 1 Laboratory of Gastrointestinal Microbiology, National Centre for International Research on Animal Gut Nutrition, Nanjing Agricultural University, Nanjing, 210095 China; 2 State Key Laboratory of Grassland Agro-Ecosystems, Key Laboratory of Grassland Livestock Industry Innovation, Ministry of Agriculture and Rural Affairs; Engineering Research Centre of Grassland Industry, Ministry of Education; College of Pastoral Agriculture Science and Technology, Lanzhou University, Lanzhou, 730000 China; 3 Department of Agriculture, Institute of Animal Nutrition, Livestock Products and Nutrition Physiology (TIER), BOKU University, Vienna, 1190 Austria

**Keywords:** Anaerobic fungi, Coumarin, Detoxication, *Melilotus officinalis*, Microbial degradation

## Abstract

**Background:**

Coumarins are toxic phytochemicals found in a variety of plants and are known to limit microbial degradation and interfere with nutrient cycling. While the degradation of coumarins by fungi has been studied in an environmental context, little is known about their degradation in the gastrointestinal system of herbivores after ingestion.

**Results:**

In this study, we investigated in vitro fermentation by microbial enrichment, transcriptome sequencing, and high-resolution mass spectrometry to evaluate the ability of rumen anaerobic fungi to degrade coumarins. The results showed that despite the low abundance of anaerobic fungi in the rumen microbiota, they were able to effectively degrade coumarins. Specifically, *Pecoramyces ruminantium* F1 could tolerate coumarin concentrations up to 3 mmol/L and degrade it efficiently via metabolic pathways involving alpha/beta hydrolases and NAD(P)H oxidoreductases within the late growth phase. The fungus metabolized coumarin to less toxic compounds, including *o*-coumaric acid and melilotic acid, highlighting the detoxification potential of anaerobic fungi.

**Conclusions:**

This study is the first to demonstrate the ability of rumen anaerobic fungi to degrade coumarin, providing new insights into the use of anaerobic fungi in sustainable agricultural practices and environmental detoxification strategies.

**Supplementary Information:**

The online version contains supplementary material available at 10.1186/s40104-025-01195-9.

## Background

Phytochemicals include secondary plant compound, which are natural chemicals with medicinal applications. However, they can play a critical role by adversely affecting healthy humans through direct contact or transfer and deposition in animal products [1]. Among the phytochemicals, coumarins are known for their phytotoxic potential, especially furocoumarin for its hepatotoxic and phototoxic activities [2, 3]. Structurally, coumarins belong to a class of phenolic phytochemicals that share the basic structure of simple coumarins consisting of a fused benzene and pyrone ring [4]. In the literature, *Melilotus officinalis* (yellow sweet clover), a legume plant known for its high yield and digestible nutrients, is used. The coumarin content in *Melilotus officinalis* can range from 0.06% to 0.753% of dry matter (DM) [5]. The characteristic odour of coumarins would result in poor palatability for ruminants and thus affect their feed intake [6]. Furthermore, when the coumarin in *Melilotus officinalis* is metabolized to dicoumarol, ruminants consuming it may develop hemorrhagic disorders [7, 8]. The conversion of coumarins into less toxic compounds, such as *o*-coumaric acid and melilotic acid, helps to reduce the risk to livestock and makes plants such as sweet clover safer for consumption. For example, coumarin appears to be the critical factor limiting the use of *Melilotus officinalis*, an annual or biennial herb of the legume family in ruminant rations [6]. Physicochemical methods such as the O_3_/percarbonate system and subcritical water oxidation are effective methods to remove coumarin contamination, but undoubtedly increase the technical difficulty and cost of the treatment process by a large margin [9, 10]. As a result, these physicochemical approaches are inefficient for the pre-treatment of large quantities of feed.

Microbial treatment such as ensilage is an environmentally friendly and economical approach to biodegrade the antinutritional factors and improve the nutritional value of forages [11]. Recent studies have mainly focused on exploring environmental microbes for coumarin degradation, such as *Pseudomonas* species [12], *Aspergillus* species [13], and *Saccharomyces cerevisiae* [14]. However, few studies have considered coumarin degradation by gastrointestinal microbes, despite the widespread presence of microbial toxin degradation in the foregut and hindgut of herbivores [15]. In fact, symbiotic gastrointestinal microbes possess an even greater diversity of detoxification responses than environmental microbes or mammalian hosts due to the selective pressures of domestic mammals, particularly herbivores [15]. Herbivores naturally consume phytochemical-rich diets and have evolved greater tolerance to toxic plant secondary metabolites than non-herbivores due to the detoxifying effects of their gastrointestinal microbiota [16]. For example, the rumen microbiota of lambs were able to convert the toxic cyanide in cassava leaves to a less toxic thiocyanate [17]. However, this ability of the gastrointestinal microbiota is limited by the concentration of the phytotoxin ingested by the animal.

Anaerobic fungi (Neocallimastigomycetes) are important contributors to the degradation of plant fiber in the rumen. They have the ability to degrade cellulose and hemicellulose, and there is some evidence that they may also play a role in modifying lignin in lignocellulosic biomass under anaerobic conditions [18]. The degradation efficiency of cellulose by rumen anaerobic fungal consortia is comparable to that of the total rumen microbiota [19]. *Pecoramyces*, a key genus within this group, has been identified as a critical microorganism for fiber degradation in the rumen. It is one of the core microbes in the rumen microbiota and is present in most ruminants regardless of host genetics and diet composition [20]. Previous research reported that the rumen anaerobic fungus *Piromyces* sp. FNG5 was tolerant to several phenolic monomers up to a level of 5 mmol/L, and more than 60% of these phenolics in the culture medium disappeared after 14 d of incubation [21], suggesting that rumen anaerobic fungi also have the potential to degrade specific phenolics. It is worth noting that coumarin is also a type of phenolic monomer, but there is still a lack of information on its degradation rate by rumen fungi, especially *Pecoramyces*. For coumarin, a previous study reported that coumarin (5% of total solids) inhibited biogas production and its levels were dramatically reduced during the anaerobic co-digestion of grass silage and cattle manure [22]. While this study investigated the response of the bacterial community to coumarin-rich plants, it remains unclear how fungi, particularly rumen anaerobic fungi, contribute to the degradation of coumarin in *Melilotus officinalis*.

This study hypothesized that rumen microbes capable of adapting to and degrading coumarins may be closely related to anaerobic fungi, despite their relatively low abundance. *Pecoramyces ruminantium* F1, a strain selected for its proven efficiency in plant degradation [23, 24], was used as the fungal model in this research. Because simple coumarin is the basic structure of coumarin compounds, this compound was selected as the sole phytochemical in this study. By investigating the mechanisms by which these fungi tolerate and degrade coumarin under rumen conditions, this research provides new insights into the metabolic pathways activated in response to phenolic stress caused by coumarin. This study is the first to investigate the potential of anaerobic fungi to degrade coumarin, providing new insights into the degradation of plant toxins using rumen microbial resources.

## Materials and methods

### Preparation of microbial inoculum and enrichment of rumen fungal consortia

The microbial inoculum was collected from the rumen fistula of 3 Hu lambs (6 months old) with an average body weight of 33 kg. All of these lambs had free access to drinking water and a diet formulation consisting of 30.0% maize, 40.0% alfalfa hay, 24.5% wheat bran, 2.5% zeolite powder, 2.5% mineral and vitamin premix (vitamin A, 70 KIU; vitamin D_3_, 8 KIU; vitamin E, 50 mg; CuSO_4_, 325 mg; ZnSO_4_, 750 mg; FeSO_4_, 550 mg; and MnSO_4_, 600 mg per kg) and 0.5% sodium bicarbonate on a DM basis, fed ad libitum with no daily weight gain target. A basal culture medium containing 1% glucose (w/v) as a carbon source, but free of antibiotics, was prepared for the rumen microbes according to Medium C described by Davies et al. [25].

Rumen fungal consortia were obtained using the basal culture medium containing different types of microbial inhibitors as described previously by Peng et al. [19]. Briefly, 2-bromoethanesulfonate was used to inhibit the growth of ruminal methanogens at a final concentration of 10 mmol/L, while penicillin and streptomycin were both used to inhibit the growth of ruminal bacteria at a final concentration of 1,600 U/mL and 2,000 U/mL, respectively. The enriched consortia were subcultured five times, with 3 d for fungi due to their slow growth rate. During the passages, 10 mL of inoculum was mixed with 90 mL of fresh Medium C and then cultured in a 180 mL serum bottle at 39 °C.

### Preparation and in vitro fermentation of *Melilotus officinalis* using whole rumen microbiota or enriched rumen fungal consortia

The *Melilotus officinalis* used as the substrate for in vitro fermentation was obtained from Lanzhou University and contained 17.7% cellulose, 16.9% hemicellulose, 6.67% acid detergent lignin, 20.6% crude protein, and 0.96% coumarin. The whole above-ground part of the *Melilotus officinalis* plant, including stems, leaves, and flowers, was collected during the early flowering stage in summer. To remove potential contaminants such as soil and dust, the plant material was washed three times with deionized water at room temperature. It was then dried at 55 °C for at least 12 h in a blast drying oven (DHG-9245A, Shanghai Youyi Instrument Co., Ltd., Shanghai, China) until a constant weight was reached. After drying, the sample was ground using a knife mill to pass through a 1-mm mesh sieve and stored at room temperature until required for fermentation studies.

The 10 mL inoculum of whole rumen microbes or fungal consortia was cultured with a 90-mL culture medium, each with 1 g DM sample of *Melilotus officinalis* as substrate. The initial culture medium contained 10^7.05^ anaerobic fungi/mL in the enriched rumen fungi treatment, 10^7.17^ anaerobic fungi/mL and 10^10.87^ bacteria/mL in the whole rumen microbiota treatment. The enriched fungi treatment was considered an “enriched” due to the use of microbial inhibitors (such as 2-bromoethanesulfonate, penicillin, and streptomycin) to suppress bacterial and archaeal growth (as described in Preparation of microbial inoculum and enrichment of rumen fungal consortia), although bacterial concentrations were not directly measured in the enriched fungal inoculum. Both blanks without substrates or inoculum were included to adjust for gas production and DM disappearance of *Melilotus officinalis*. Gas production was measured in serum bottles. Serum bottles of 180 mL volume were used as the vessels for microbial incubation with 4 replicates for each treatment. Microbial consortia for inoculation of both whole rumen and fungal consortia were incubated at 39 °C in a constant temperature incubator without agitation. Gas production from the whole rumen microbiota was measured at 3, 6, 9, 12, 24, 36, and 48 h. For the fungal consortia, gas production was recorded every 8 h (8, 16, 24, 32, 40, 48, 56, 64, 72, 80, 88, and 96 h) over a 96 h incubation period. The difference in incubation time is mainly due to microbial growth rates, which are faster for the whole rumen microbiota, and the resulting plateau phase for gas production. The culture medium of the whole rumen microbiota (48 h) and fungal consortia (96 h) was collected for analysis of fungal metabolism, microbial crude protein, and microbial copy number. Substrate residues were obtained by filtering through the nylon cloth with a pore size of 48 μm to determine the DM disappearance of *Melilotus officinalis*. To minimize the effect of microbial attachment on the degradation rate, the substrate was washed three times with PBS before filtration.

### Genomic DNA of ruminal microbes in culture medium

Genomic DNA was extracted using the commercial kits from Tiangen Biochemical Technology Co., Ltd. (No. DF328, Beijing, China). Its concentration and quality were determined using a Nanodrop 2000 spectrophotometer (Thermo Fisher Scientific, MA, USA). According to the literature [26], quantitative PCR of high-quality microbial DNA, defined by a A_260_/A_280_ ratio between 1.7 and 2.0, was performed using a 7500 real-time PCR system (Applied Biosystems, Foster City, CA, USA). The targeted primers for bacteria were F: 5′-CCTACGGGAGGCAGCAG-3′ and R: 5′-ATTACCGCGGCTGCTGG-3′ [27], and for anaerobic fungi were F: 5′-GAGGAAGTAAAAGTCGTAACAAGGTTTC-3′ and R: 5′-CAAATTCACAAAGGGTAGGATGATT-3′ [26]. Total copy numbers of bacteria or anaerobic fungi were calculated using a standard curve generated with linearized pMD18-T plasmids (Takara Bio Inc., Shiga, Japan) as published elsewhere [28].

### Preparation of anaerobic fungal supernatant from *Melilotus officinalis* silage

To obtain the anaerobic fungal supernatant silage additive, rumen fluid was collected from the rumen fistula of the aforementioned Hu lambs and prepared according to the previously published method [29]. Briefly, to obtain the anaerobic fungal supernatant, rumen fluid collected from the rumen fistula was supplemented with antibiotics (50 µg/mL chloramphenicol, 20 µg/mL streptomycin, 50 µg/mL penicillin) and anaerobically cultured in Medium C [25] at 39 °C for 4 d, using 4 g/L beechwood xylan (P-XYLNBE-10G; Megazyme, Wicklow, Ireland) as a carbon source. During this period, the presence of bacteria or methanogens was monitored by microscopic observation and methane measurement. Prior to the ensilage process, the anaerobic fungal supernatants from all serum bottles were combined and thoroughly mixed.

The experimental group was sprayed with 10 mL of the anaerobic fungal mixture supernatant and the control group was sprayed with 10 mL of deionized water. The bag was stored under vacuum at room temperature away from light for a total of 60 d. Eight sampling times (1, 3, 5, 7, 15, 30, 45 and 60 d) were established in the experiment, with a total of 32 bags in each group. At each time point, 4 vacuum bags were opened for destructive sampling and analysis.

To compare the effects of anaerobic fungal supernatant silage additives, the corresponding indicators were determined. The pH was measured directly using a calibrated pH meter (Ecoscan pH 5, Singapore) inserted into the silage. The concentration of lactate was determined using an Agilent 1220A Infinity liquid chromatograph equipped with a ZorbaxSB-Aq column (250 mm length × 4.6 mm inner diameter, 5 μm particle size, Agilent Technologies) according to a previously published method [23]. The concentration of NH_3_-N was determined according to the method of Weatherburn et al. [30]. Crude protein (CP) was determined by the Kjeldahl nitrogen determination method [31]. Neutral detergent fiber (NDF) and acid detergent fiber (ADF) contents were determined using the ANKOM A200I fiber analyzer [32]. Specifically, the substrate was processed in an ANKOM fiber analyzer (ANKOM A200I, Ankom Technology, NY, USA) and mixed with a neutral detergent solution. Thermostable α-amylase (2 × 10^4^ U/mL, Shanghai Yuanye Biotechnology Co., Ltd., Shanghai, China) was added at a concentration of 2 mL/L of neutral detergent solution, and the mixture was incubated at 100 °C for 75 min before drying. The remaining residue represented the NDF content. For ADF determination, the NDF residue was treated with an acid detergent solution in the same analyzer, incubated at 100 °C for 60 min, and then dried. Starch content was determined using the starch kit (A148-1-1, Nanjing Jiancheng Bioengineering Institute, China). The water-soluble carbohydrate (WSC) content in the samples was determined by the anthrone-sulfuric acid colorimetric method [33]. The coumarin content was determined as described by Zhao et al. [34].

### Interaction between glucose and coumarin on the growth of *Pecoramyces ruminantium* F1

*Pecoramyces ruminantium* F1 was selected as the rumen-derived fungal strain model for this study because its efficient degradation capacity has been widely demonstrated in previous studies [23, 24]. The anaerobic fungus *P. ruminantium* F1 used in the experiment was obtained from a previous study [24]. *P. ruminantium* F1 was routinely maintained in the Medium C according to the method described by Cheng et al. [35]. Chloramphenicol was also included in the cultures at a final concentration of 50 mg/L to prevent bacterial contamination [35].

The coumarin concentration was based on the results of Cansunar et al. [36] that glucose uptake by anaerobic rumen fungi in vitro was inhibited by 5 mmol/L or more of coumarin. A two-factor experiment was performed with two levels of glucose (i.e., 0 and 55 mmol/L) and coumarin (i.e., 0 and 5 mmol/L) in the culture medium for the *P. ruminantium* F1 (*n* = 4). The same volumes of deionized water or dimethyl sulfoxide (DMSO) were added when the glucose or coumarin concentrations were 0 mmol/L, respectively. Before the start of the experiment, glucose was dissolved in deionized water for the 50% glucose solution, while coumarin (Shanghai Macklin Biochemical Co., Ltd., Shanghai, China) was dissolved in DMSO to prepare a 1 mol/L stock. The culture medium without the fungal inoculum was used as a blank (*n* = 4) to adjust the gas output of each treatment. Gas outputs were determined every 8 h during the 7 d incubation period. The culture medium was sampled at mid-stage (d 4) and late stage (d 7) for analysis of fungal metabolites and the utilization of coumarin and glucose by *P. ruminantium* F1. The culture medium after inoculation on d 0 was also sampled and used as a baseline.

The glucose concentration in the medium samples was analyzed using a commercial kit (No. A154-1-1) purchased from Nanjing Jiancheng Bioengineering Institute (Jiangsu, China). For the coumarin concentration, the medium samples were completely mixed with equal volumes of methanol and determined using a 1220A Infinity liquid chromatograph equipped with a ZorbaxSB-Aq column (Agilent Technologies) according to previously published methods [34]. Utilization rates of glucose and coumarin by *P. ruminantium* F1 were calculated on the basis of parallel culture vials containing the corresponding substances but without the fungal inoculum (*n* = 4). These uninoculated vials were sampled at the same time points to allow for possible non-biological degradation of the substrates, which could then be corrected in the degradation calculations.

### Dose effects of coumarin on the growth of *Pecoramyces ruminantium* F1

Five levels of coumarin were included in the culture medium containing 1% glucose, 1.0, 2.0, 3.0, 4.0 and 5.0 mmol/L, respectively, to study the dose effects of coumarin on the growth of *P. ruminantium* F1. Culture medium without coumarin solution but with the addition of an equal volume of DMSO was used as a blank. Each treatment included 4 replicates to determine gas production every 8 h during the 7 d incubation. On d 7, the medium samples from each treatment were collected for analysis of fungal metabolites.

### Ability of enzymes secreted by *Pecoramyces ruminantium* F1 to degrade coumarin

The culture medium containing 1% glucose (w/v) was collected after incubation of *P. ruminantium* F1 for 3 d (4 replicates). Cell-free supernatant was obtained by centrifugation of the medium sample at 15,000 × *g*, 4 °C for 10 min and filtration through 0.22 μm pore size membranes. Next, 25 mL of supernatant from each replicate was immediately incubated with 100 μL coumarin solution (1 mol/L stock) at 39 °C, while another was treated in the same way after inactivating the enzymes in the culture medium by heating at 103 °C for 4 h and then cooling. After 24 h of incubation, the coumarin concentration in the medium samples was determined to calculate the average degradation rate, based on the coumarin concentration in the heat-treated medium, which provides a general estimate of the degradation but does not take into account the kinetic order of the reaction.

### RNA sequencing and data analysis of *Pecoramyces ruminantium* F1 in response to coumarin

Two coumarin concentrations (i.e., 0 and 3 mmol/L) were used as the treatments based on the dose effects of coumarin on growth. At the first log growth (48 h) of incubation, determined by taking the logarithm of the microbial gas production curve, fungal cells were harvested for the RNA-seq analysis of *P. ruminantium* F1. Sediments were washed twice with phosphate buffer and stored at −80 °C for later analysis. Total RNA in the samples was extracted using TRIzol. Samples were removed from liquid nitrogen, thawed on ice, and ground to a powder. TRIzol was added, and the mixture was vortexed and centrifuged at 4 °C and 10,000 × *g* for 5 min. The resulting supernatant was collected, and 1 mL of the lysate was mixed with 200 μL of chloroform/isoamyl alcohol (24:1), followed by vigorous inversion. After a second round of centrifugation, the supernatant was collected, and an equal volume of isopropanol was added. The mixture was stored at −20 °C for 1 h and then centrifuged at 10,000 × *g* and 4 °C for 20 min. The supernatant was carefully removed, and the RNA pellet was washed with 75% ethanol. A further centrifugation step was performed, and the supernatant was removed. The pellet was dried briefly at room temperature and then dissolved in RNase-free water. RNA concentration was measured using the NanoDrop system (NanoDrop, Madison, USA). The mRNA was purified from total RNA using oligo(dT)-bound magnetic beads and then fragmented into smaller pieces using divalent cations under elevated temperature. The first and second strands of cDNA were synthesized by random hexamer primed reverse transcription. The cDNA library was subjected to end repair and ligation with adapters, followed by amplification. The size and concentration of the library fragments were determined using an Agilent 2100 Bioanalyzer. Paired-end sequencing of 150 bp length was performed on the BGISEQ-500 platform (BGI, Shenzhen, China).

After sequencing, the raw reads were filtered using SOAPnuke software to remove adapters and low-quality reads according to a previously published method [37]. The clean data were merged using Trinity software (v2.14.0) and then annotated using the Kyoto Encyclopedia of Genes and Genomes (updated on Nov 1^st^, 2022) by blasting the sequences using Trinotate (v3.2.2). Carbohydrate-active enzymes (CAZymes) within genes were annotated using the tools of HMMER (v3.3.2), eCAMI [38] and Diamond (v4.5.515) tools with the CAZy database (updated on Nov 15^th^, 2022; http://www.cazy.org). CAZymes identified as the same by at least two of these tools were considered effective. Gene expression was quantified using the RESM tool (v1.3.3) and normalized between samples.

### Growth and metabolic analysis of *Pecoramyces ruminantium* F1

Gas emissions during incubation were measured using a pressure transducer (Ankom RFS, Ankom Technology, NY, USA) [39]. Hydrogen concentrations in gas samples were determined on a 7890B gas chromatograph equipped with a thermal conductivity detector and a GC column packed with Porapak Q (No. G3591-80135, 0.91 m length × 2 mm inner diameter, Agilent Technologies, CA, USA) according to a previously published method [23]. Cumulative gas and hydrogen emissions were calculated [35]. Concentrations of short-chain fatty acids and lactate in the culture medium were determined using an Agilent 1220A Infinity liquid chromatograph equipped with a ZorbaxSB-Aq column (250 mm length × 4.6 mm inner diameter, 5 μm particle size, Agilent Technologies) according to a previously published method [23]. Ethanol concentration in the medium was determined using an Agilent 7890B gas chromatograph (Agilent Technologies) equipped with a capillary GC column (No. 24107, 30 m length × 0.25 mm inner diameter, Merck KGaA, Darmstadt, Germany) according to previously published method [40].

Culture fluid from *P. ruminantium* F1 treated with 3 mmol/L coumarin was collected daily during incubation for analysis of coumarin-degrading metabolites (*n* = 4). Liquid samples were stored at −80 °C prior to analysis. For metabolite extraction, a 100 μL liquid sample was mixed with 300 μL methanol and centrifuged at 10,000 × *g* for 20 min. The supernatant of each mixture (300 μL) was dried using a vacuum centrifugal concentrator. The residue was redissolved with a 2% aqueous methanol solution and passed through a 0.22-μm pore size filter to obtain the fungal metabolites. Fungal metabolites were identified using a combined ultra-high liquid chromatography and mass spectrometry (UPLC-MS, Q-Exactive Plus, Thermo Fisher Scientific, MA, USA) equipped with a Hypersil GOLD VANQUISH column (100 mm length × 2.1 mm inner diameter, 1.9 μm particle size, Thermo Fisher Scientific, MA, USA). Samples were eluted using two types of mobile phases for different detection modes: 0.1% aqueous formic acid solution (vol/vol, phase A) and methanol solution with 0.1% formic acid (vol/vol, phase B) for positive ion mode (ESI +) and 0.05% aqueous acetic acid solution (vol/vol, phase A) and methanol solution with 0.05% acetic acid (vol/vol, phase B) for negative ion mode (ESI−). The mobile phase gradient eluent was run at a flow rate of 0.3 mL/min for 0.0 to 0.5 min, 2% phase B; 0.5 to 10.0 min, 2% to 50% phase B; 10.0 to 14.0 min, 50% to 98% phase B; 14.0 to 16.0 min, 98% phase B.

A high-resolution mass spectrum was acquired in the mass range of 70.0 to 1,050.0 *m/z* at a resolution of 70,000 with an AGC target at 1e^6^. The dd-MS^2^ data were acquired at a resolution of 17,500 with an AGC target at 1e^5^. The sheath gas, auxiliary gas and sweep gas were at flow rates of 45 arb, 10 arb, and 2 arb, respectively. The capillary and auxiliary gas heater temperatures were 300 °C and 350 °C, respectively. The spray voltage was 3.5 kV for positive mode and −4.5 kV for negative mode, while the S-lens RF level was 50.0. A volume of 10 μL samples was injected for UPLC-MS analyses. Quality control samples were prepared by mixing all liquid samples and injected every 10 determinations. The raw data obtained were analyzed using Compound Discoverer (v3.3, Thermo Fisher Scientific, MA, USA) with metabolite databases (mzCloud, mzVault, and ChemSpider). Fold changes of coumarin degrading metabolites were calculated using the d 0 fluid sample as the base.

### Zoospore, biofilm, and cell weight of *Pecoramyces ruminantium* F1

At the mid-exponential stage of fungal growth (d 3), 300 μL of culture medium was collected from each serum bottle after light mixing with 3 replicates for each treatment. The medium samples were injected into a 48-well culture plate to examine the zoospores of *P. ruminantium* F1 using the DMi8 inverted microscope (Leica Microsystems, Wetzlar, Germany). Four bottles of each treatment were destructively sampled to measure fungal cell weight at mid-stage (4 d incubation) and late stage (7 d incubation). The culture medium above the biofilms was carefully collected without shaking the serum bottles, and then the biofilms were placed in the culture dishes to record the morphology of the main fungal biofilms. After observation, the biofilms were mixed with the previous culture medium. The mixture was centrifuged at 10,000 × *g* for 15 min at 4 °C to collect the mycothallus. The mycothallus was washed twice with distilled water and transferred to reweighed tubes and then lyophilized for 24 h in a FreeZone 4.5 freeze dryer (Labconco, MO, USA) to determine the dry cell weight of *P. ruminantium* F1.

### Statistical analysis

Comparisons between whole rumen microbiota and enriched rumen fungi in response to *Melilotus officinalis*, as well as comparisons between the nutritional value of anaerobic fungal supernatant ensilage and non-ensilage of *Melilotus officinalis*, were analyzed using the Student’s *t*-test in R software (v4.2.1). Interactions between glucose and coumarin on the growth of *P. ruminantium* F1 were analyzed using two-way ANOVA analysis in R software (v4.2.1). Degradation rates of glucose or coumarin at different growth stages were analyzed using the Student’s *t*-test in R software (v4.2.1). Dose effects of coumarin on *P. ruminantium* F1 were analyzed using the one-way ANOVA analysis in R software (v4.2.1) with the LSD method for multiple comparisons and the Benjamini–Hochberg method for *P*-value correction. The dose effects of coumarin were also analyzed using the linear and quadratic regression in R software (v4.2.1). The quadratic curve was fitted using GraphPad Prism (v9.4.1) with 95% confidence bands in the graph to obtain the fitting equations and degrees (*R*^*2*^). Results with *P* < 0.05 were considered as significant differences. For the transcriptome, principal component analysis was performed to compare the fungal gene composition between treatments using the OmicStudio platform (https://www.omicstudio.cn/), with ANOSIM analysis for the *P*-value and correlation coefficient *R*. Differential genes were identified using the DESeq2 package (v1.38.1) in R software (v4.2.1). The* P*-values of differential genes were adjusted using the Benjamini–Hochberg method. Genes with an adjusted *P*-value (FDR) < 0.05 and log_2_ (fold change) > 2 or < −2 were considered to be different between treatments. A volcano plot was used to visualize the differential genes using the OmicStudio platform (https://www.omicstudio.cn/), with fold change and FDR as the classification standard. Pathway enrichment analysis was performed using clusterProfiler (v4.0) [41] after annotation of fungal genes using GO and KO databases.

## Results

### Greater coumarin degradation efficiency of anaerobic fungi despite low abundance in rumen microbiota

**Fig. 1:**
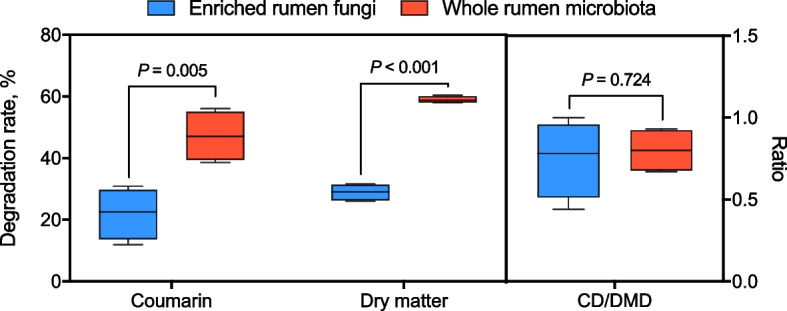
Coumarin degradation by in vitro incubation of enriched rumen fungi and whole rumen microbiota with *Melilotus officinalis* as the substrate. Two treatments both lasted until the growth plateau (i.e., 96 h for enriched rumen fungi and 48 h for whole rumen microbiota). CD/DMD represents the ratio of coumarin degradation (CD) to dry matter degradation (DMD). Within each mL of initial culture medium, enriched rumen fungi contained and average of 10^7.05^ anaerobic fungi, while the whole rumen microbiota contained an average of 10^7.17^ anaerobic fungi and 10^10.87^ bacteria

Plant secondary metabolites are natural compounds with toxic or antinutritional properties that serve as a defense mechanism against herbivores. In *Melilotus officinalis*, coumarin is the specific metabolite that results in poor palatability and inhibitory effects on gastrointestinal microbes [6, 42]. Based on the limited research on the contribution of different rumen microorganisms to degradation, this study first compared the coumarin degradation of different rumen microbial consortia to explore the rumen microbes that efficiently degrade coumarin. Unlike anaerobic fungi, rumen bacteria can more efficiently degrade a wider range of plant carbohydrates, leading to the production of greater amounts of volatile fatty acids (VFAs), including acetate, propionate, and butyrate. This results in higher gas production by the whole rumen microbiota compared to the enriched fungal consortia (Fig. S1). Furthermore, the degradation process led by the rumen bacterial consortium reached the gas production plateau in half the time compared to the fungal consortium. The whole rumen microbiota had a better ability to degrade coumarin (*P* = 0.005) and DM (*P* < 0.001) of *Melilotus officinalis* than the enriched rumen fungi (Fig. 1). However, no difference was observed in the ratio between the ratio of coumarin and DM degradation in the substrate (*P* = 0.724) by the enriched rumen fungi (10^7.05^ fungi/mL) and the whole rumen microbiota (10^7.17^ fungi/mL and 10^10.87^ bacteria/mL) (Fig. 1). Although the abundance of rumen fungi was relatively low, the degradation of coumarin in *Melilotus officinalis* did not differ significantly between the fungal enrichment and the whole microbiota inoculum, as indicated by the similar ratios of CD to DMD. In view of this observation, the rumen fungal consortium of *Melilotus officinalis* deserves further attention for its potential application as a silage additive.

### Anaerobic fungal supernatant as silage additive can reduce the interference of coumarin in *Melilotus officinalis*

With increasing incubation time in silage, the nutritional value of *Melilotus officinalis* in the anaerobic fungi group was higher than that in the control group (Fig. S2). Dry matter gradually decreased in both groups, with a significant difference between the experimental group and control group on d 3 (*P* < 0.05). In addition, significant differences in CP concentration between the two groups appeared on the 5^th^ day of silage and persisted until the end, while the difference in WSC concentration gradually decreased as the ensiling process continued. The NDF, ADF, and starch contents of the two groups differed at some time points rather than throughout the silage incubation period (*P* < 0.05).

Nutrient concentrations and coumarin degradation in *Melilotus officinalis* were higher in the anaerobic fungi group than in the control group (Fig. S3). In particular, the coumarin degradation rate was significantly higher in the experimental group from the first day of ensiling (*P* < 0.05), and this difference remained significant throughout the entire ensiling period. This suggests that anaerobic fungal supernatant, when used as a silage additive, can effectively degrade coumarins while preserving the nutritional value of *Melilotus officinalis*, which is consistent with the in vitro rumen fermentation results mentioned above. In addition, this study also examined the changes in pH and lactate production within the silage environment (Fig. S4). The pH of the control group decreased significantly throughout the silage process compared to the experimental group, and similarly, its lactate concentration was higher than that of the experimental group.

Based on the above results, ruminal anaerobic fungi have the potential to effectively degrade coumarin. Further experiments will focus on the specific mechanisms by which ruminal anaerobic fungi tolerate and detoxify phenolic coumarins, as *Pecoramyces* is widely distributed in the rumen and feces of ruminants, independent of host genetics and diet composition [20]. Therefore, *P*. *ruminantium* F1, which has been widely demonstrated to have efficient degradation capacity [23, 24], was selected as a rumen-derived fungal strain model for further experiments.

### High concentrations of coumarin inhibited the growth of *Pecoramyces ruminantium* F1 and could not serve as its energy source

**Fig. 2:**
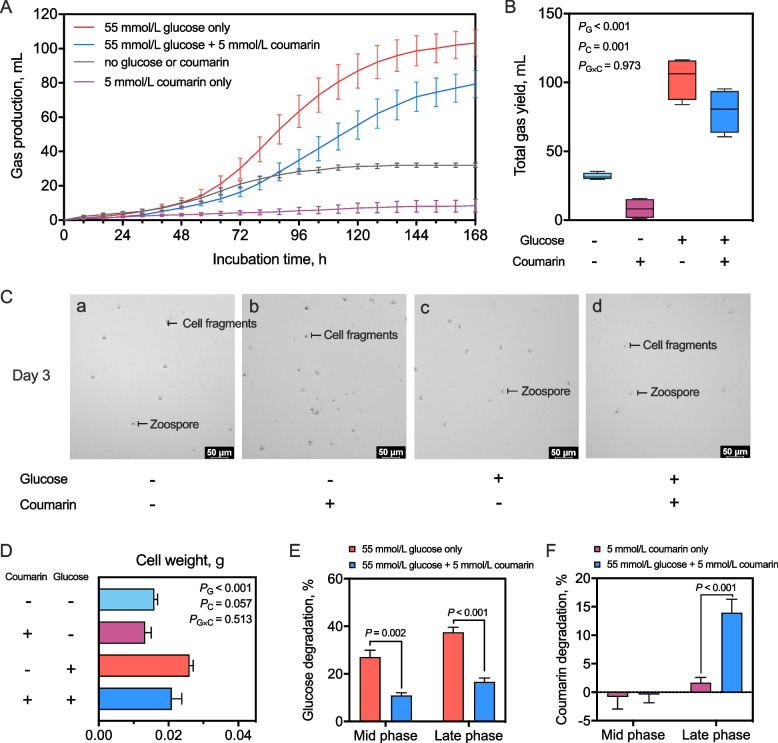
Effects of coumarin (5 mmol/L) and glucose (55 mmol/L) on the gas production and fungal growth of *P*. *ruminantium* F1. **A** and **B** Curves of total gas production. **C** Microscopic examination of fungal zoospore at mid-exponential phase (Day 3). **D** Cell weight of *P*. *ruminantium* F1 at late phase (Day 7). **E** and **F** Degradation of glucose and coumarin in the culture fluid

The above results indicate that enriched rumen anaerobic fungi have a strong potential for coumarin degradation and applications in the field of silage production. Coumarins are the phenolics that could be used as the sole carbon source for the growth of some microbes, such as *Pseudomonas* species [34]. However, it remains unclear whether anaerobic fungi can utilize coumarin as an energy substrate like *Pseudomonas* species. To explore this question, this study attempted to compare two sole carbon sources (i.e., coumarin and glucose, the latter being a common carbon source for anaerobic fungal growth) on the growth and gas production of an axenic culture of *P*. *ruminantium* F1. The results showed that 5 mmol/L coumarin significantly decreased the gas production of *P*. *ruminantium* F1 whether glucose was added or not (*P* = 0.001) and showed no interaction with the addition of 55 mmol/L glucose (*P* = 0.973) (Fig. 2). The reason for the lower fungal growth rate could be attributed to two aspects. First, the efficiency of glucose utilization by *P*. *ruminantium* F1 was inhibited by coumarin (*P* < 0.05, Fig. 2E). Second, coumarin depressed the zoosporogenesis of *P*. *ruminantium* F1, resulting in a decreased number of zoospores and increased cell fragments (Fig. 2C), and consequently reduced the cell weight of *P*. *ruminantium* F1 at the late growth stage (d 7; *P* < 0.001, Fig. 2D). Because the zoospores are the initial growth stage of anaerobic fungi and could be the indicator of fungal abundance in vitro [43], coumarin could depress zoosporogenesis to inhibit the growth of *P*. *ruminantium* F1. Thus, high concentrations of coumarin inhibited the growth of *P*. *ruminantium* F1 resulting in a broken fungal biofilm and decreased concentrations of fungal metabolites, including acetate, lactate, formate, and ethanol (Fig. S5). For coumarin degradation, the results of Fig. 2F showed that the culture medium containing 55 mmol/L glucose caused a greater coumarin degradation than that without adding glucose at the late growth stage of *P. ruminantium* F1 (*P* < 0.001). This result indicates that coumarin degradation occurred in the late growth phase rather than in the mid-growth phase of *P*. *ruminantium* F1.

### Rumen anaerobic fungus *Pecoramyces ruminantium* F1 has a tolerance to coumarin with up to 3 mmol/L concentration

**Fig. 3:**
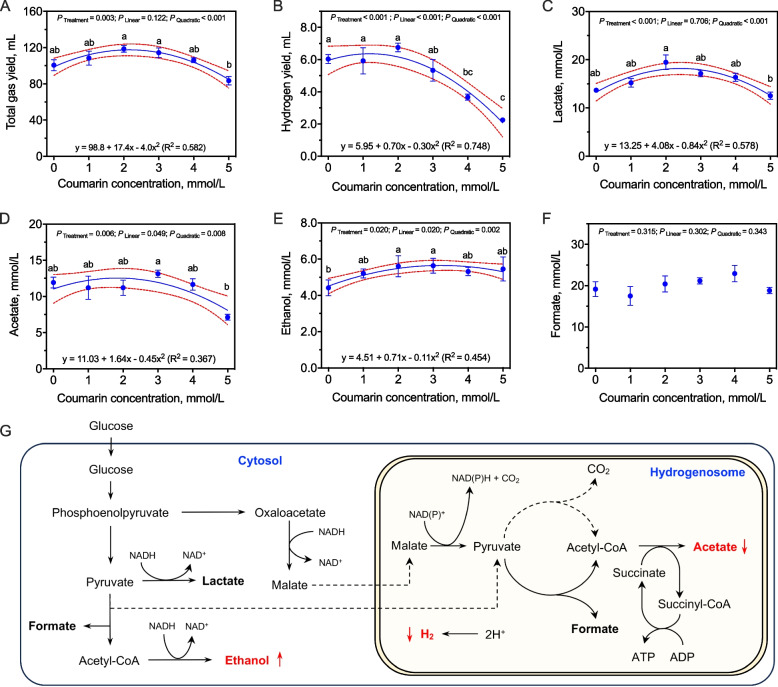
Dose effects of coumarin on the metabolism of *P*. *ruminantium* F1. **A** and **B** Yields of total gas and hydrogen. **C–F** Concentrations of major metabolites of *P*. *ruminantium* F1, including lactate, acetate, ethanol, and formate. **G** Schematic diagram of the changes in energy metabolism pathways of *P*. *ruminantium* F1

A previous study reported that anaerobic fungi had a high tolerance to phenolics, with up to 5 mmol/L by *Piromyces* species isolated from wild nil gai [21]. In the present study, a 5 mmol/L concentration of coumarin significantly inhibited the growth of *P*. *ruminantium* F1 (Fig. 2). Therefore, the tolerance concentration of coumarins to anaerobic fungal *P*. *ruminantium* F1 was further studied. The dose effects of coumarin showed that adding coumarin affected the total gas yield (*P* < 0.001, *R*^2^ = 0.582) and H_2_ yield (*P* < 0.001, *R*^2^ = 0.748) of *P*. *ruminantium* F1 in a quadratic manner (Fig. 3). Except for gas yield, the primary metabolites, including formate, lactate, acetate, and ethanol, were also produced during the axenic culture of *P*. *ruminantium* F1 using glucose as the substrate [24]. Results also showed that the coumarin addition significantly affected the concentrations of lactate (*P* < 0.001), acetate (*P* = 0.006), and ethanol (*P* = 0.020) but had no effects on formate concentration (*P* = 0.315, Fig. 3) during the incubation of *P*. *ruminantium* F1. The tolerance of the anaerobic fungus *P. ruminantium* F1 to coumarin appears to be limited to 3 mmol/L. At higher coumarin concentrations, gas production by *P*. *ruminantium* F1 and the formation of various water-soluble metabolites during fermentation are significantly inhibited.

### Coumarin induces changes in metabolic pathways and genes encoding carbohydrate-active enzymes of *Pecoramyces ruminantium* F1

Compared with the heat-treated inactivated culture medium, the average coumarin degradation rate was 0.16 mmol/L/h (standard deviation = 0.028) when using the cell-free culture medium of *P. ruminantium* F1. This result suggested that *P. ruminantium* F1 could secrete extracellular enzymes to degrade coumarin. Because *P*. *ruminantium* F1 can tolerate 3 mmol/L coumarin in the culture environment, the specific transcriptomic and metabolic responses under this condition were investigated in detail. For transcripts, a total of 18,386 genes of *P. ruminantium* F1 were obtained by RNA-seq analysis in the present experiment. Results of principal component analysis showed that 3 mmol/L coumarin induced a significant transcriptomic change of *P. ruminantium* F1 (*P* = 0.009, *R* = 0.676, Fig. S6). Compared to the control, the addition of 3 mmol/L coumarin altered 13.9% of all the expression of genes of *P. ruminantium* F1 (FDR < 0.05), of which 52.2% of these genes were upregulated (1,331 genes). To specifically investigate the genes substantially induced by coumarin (FDR < 0.05, log_2_FC > 2 or < −2), 390 up-regulated genes and 136 down-regulated genes were used for further analysis. These substantially altered genes resulted in the enrichment of 2 KEGG orthologs, 7 biological processes, 2 molecular functions, and 10 cellular components (Fig. S7).

**Fig. 4:**
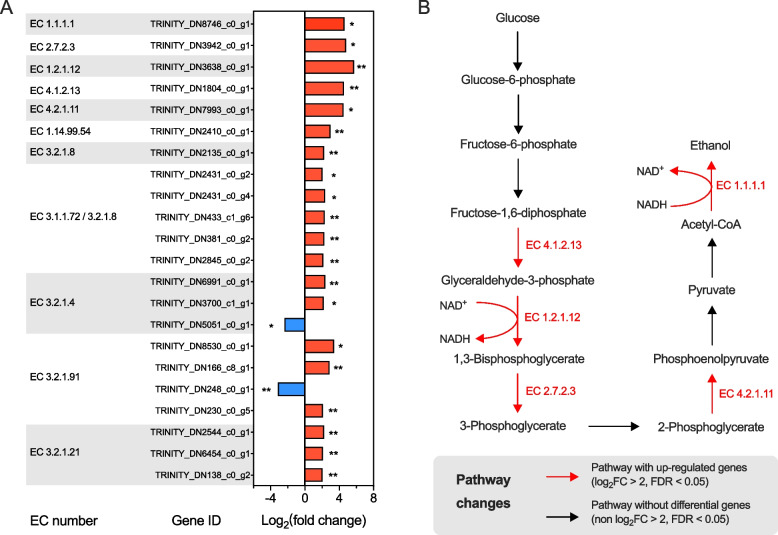
Changes in the genes encoding carbohydrate metabolism in *P*. *ruminantium* F1 and related pathways induced by the addition of 3 mmol/L coumarin. **A** Differential genes encoding carbohydrate metabolism. **B** Partial pathway changes in glucose utilization. Symbols * and ** indicate FDR < 0.05 and < 0.01, respectively. Other notes: EC 1.1.1.1, alcohol dehydrogenase; EC 2.7.2.3, phosphoglycerate kinase; EC 1.2.1.12, glyceraldehyde 3-phosphate dehydrogenase (phosphorylating); EC 4.1.2.13, fructose-bisphosphate aldolase; EC 4.2.1.11, enolase; EC 1.14.99.54, lytic cellulose monooxygenase (C1-hydroxylating); EC 3.2.1.8, endo-1,4-β-xylanase; EC 3.1.1.72, acetylxylan esterase; EC 3.2.1.4, endoglucanase; EC 3.2.1.91, cellobiohydrolase; EC 3.2.1.21, β-glucosidase

According to the KEGG database, most of the differential genes could be classified into the ribosome pathway, metabolic pathways, biosynthesis of secondary metabolites, and the pathways related to carbohydrate metabolism (Fig. S6), which was consistent with above finding that the addition of coumarin altered the pathways of fungal carbohydrate metabolism (Fig. 3G). Except for the genes encoding for fiber-degrading enzymes, the results of Fig. 4 also showed that 3 mmol/L coumarin upregulated several genes encoding for the glucose metabolic enzymes including fructose bisphosphate aldolase (EC 4.1.2.13), glyceraldehyde 3-phosphate dehydrogenase (phosphorylating, EC 1.2.1.12), phosphoglycerate kinase (EC 2.7.2.3), etc. The increased expression of these genes, especially alcohol dehydrogenase (EC 1.1.1.1, log_2_FC = 4.59, FDR = 0.017), indicated that the metabolic pathway from glucose to ethanol was enhanced by 3 mmol/L coumarin (Fig. 4B), since alcohol dehydrogenase is the enzyme that catalyzes acetyl-CoA to ethanol in the cytosol of anaerobic fungi [44]. This result could also explain the linear increase in ethanol in the culture fluid caused by the increased coumarin dose as the pathway changes shown in Fig. 4B.

**Fig. 5:**
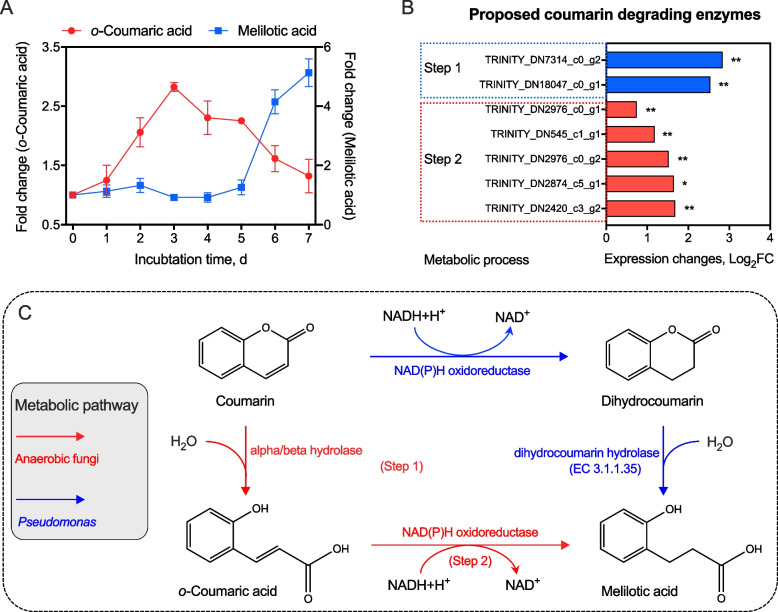
Proposed metabolic pathway of coumarin by *P*. *ruminantium* F1 incubated with 3 mmol/L coumarin. **A** Fold changes of coumarin degradation products during incubation. **B** Gene expression of proposed coumarin degrading enzymes. **C** Proposed coumarin metabolic pathway of *P*. *ruminantium* F1 compared with *Pseudomonas* spp. Step 1 included the genes encoding for alpha/beta hydrolases, while step 2 included the genes encoding for NAD(P)H oxidoreductases. The symbols * and ** represented FDR < 0.05 or < 0.01, respectively

In addition, in order to understand the specific metabolic pathway of anaerobic fungi in the degradation of coumarin, this study used UPLC-MS to identify the major metabolites during the degradation process. UPLC-MS analysis detected two primary coumarin metabolites (*o*-coumaric acid and melilotic acid) in the culture medium, and their concentrations changed over time (Fig. 5A). The relative concentration of *o*-coumaric acid peaked at d 3, while melilotic acid subsequently increased after the degradation of *o*-coumaric acid by d 5.

## Discussion

*Melilotus officinalis* is highly adaptable to extreme environmental conditions such as cold and drought, and it can thrive in moderately saline soils where other traditional forage legumes struggle to survive [45]. Studies have shown that *Melilotus officinalis* has a significantly higher yield and superior nitrogen fixation compared to *Medicago sativa*. However, its widespread use as hay or silage has been limited due to its high coumarin content. Previous studies reported that cattle gastrointestinal microbes could effectively degrade coumarin in the anaerobic co-digestion of grass silage and cattle manure [22]. Therefore, the goal of this study was to identify rumen microbial resources capable of efficiently degrading coumarins, with the aim of enhancing the nutritional value of *Melilotus officinalis*. This study highlights the superior ability of rumen anaerobic fungi in coumarin degradation, providing a promising microbial solution to improve the nutritional value of *Melilotus officinalis*. This finding was consistent with previous studies that reported that rumen anaerobic fungi had strong tolerance and detoxification of specific phenolics [21]. Other research has confirmed that rumen anaerobic fungi are highly efficient in degrading different types of substrates [19]. This efficiency is due to their physical invasion via rhizoids and the secretion of a substantial number of fiber-degrading enzymes. Effective research and utilization of anaerobic fungi and the enzymes they produce will be crucial to promote the use of unconventional forages such as *Melilotus officinalis*.

The results of this study demonstrate the efficacy of anaerobic fungal supernatant as a silage additive in improving the nutritional quality of *Melilotus officinalis*. The experimental group consistently showed higher nutrient retention and coumarin degradation rate compared to the control group. The significant difference in CP concentration that appeared on d 5 and persisted throughout the ensiling process is consistent with previous findings by Muck et al. [46], who reported that the incorporation of microbial additives can increase CP levels in silage by promoting more efficient fermentation processes. In addition, while the WSC concentrations gradually converged between the groups, the experimental group maintained a superior nutrient profile, supporting the conclusions of Hartinger et al. [29] who found no effect of anaerobic fungal treatment on WSC after ensiling. The most notable finding was the rapid and sustained degradation of coumarin in the anaerobic fungal group from the first day, indicating the potential of anaerobic fungi to detoxify phenolic compounds while maintaining silage quality. Although there have been many previous studies on the use of anaerobic fungal supernatant as a silage additive, this study is the first to focus on its effect on coumarin degradation. In addition, lactic acid bacteria and anaerobic fungi may have a synergistic effect during the ensiling process. Lactic acid bacteria can provide an ideal growth environment for anaerobic fungi by lowering pH and producing lactic acid (reducing mold and other harmful bacteria) [46]. Anaerobic fungi can effectively break down plant fibers with fiber-degrading enzymes, releasing glucose that can be used by lactic acid bacteria, creating a virtuous cycle.

In the present study, we observed that treatment with the fungal supernatant inhibited the normal pH decrease typically seen during silage fermentation. This finding suggests that the fungal inoculum may alter the fermentation process in a way that limits the production of acids that are normally responsible for silage acidification. Such an effect could be considered a negative result, as effective silage fermentation generally relies on a rapid pH drop to prevent spoilage by undesirable microbes. The reduced acidification observed with the fungal treatment warrants further investigation into the specific mechanisms by which fungal metabolites might influence pH regulation in the silage. Unfortunately, we did not test for acetic acid or other organic acids in the silage. This limitation may explain the observed pH patterns and highlights the need for further research into the broader profile of fermentation products. Future studies should include measurements of acetic acid, as well as other VFAs, to fully characterize the fermentation dynamics and provide a more complete understanding of the microbial interactions occurring during silage fermentation.

Because gas production could indicate the growth and amount of anaerobic fungi [23], the result in this study suggested that high levels of coumarin would inhibit the growth of *P*. *ruminantium* F1, which is consistent with previous studies [36]. This phenomenon suggests that certain phenolic compounds can exert inhibitory effects on microbial growth in anaerobic systems [21], although the mechanisms may differ between species. Zoospores represent the initial growth stage of anaerobic fungi and are essential for colonization and biofilm formation [18]. Inhibition of zoosporogenesis likely disrupts these early growth processes, resulting in reduced fungal biomass and weakened biofilm integrity. Studies of other phenolics have similarly noted their disruptive effects on fungal zoospore release and motility, suggesting that coumarin may act through a comparable mechanism [47]. The results of this study suggest that while *P. ruminantium* F1 can degrade coumarin, it may require an additional carbon source such as glucose to do so efficiently, particularly in later growth stages.

The tolerance of the anaerobic fungus *P. ruminantium* F1 to coumarin appears to be limited to 3 mmol/L, which is partially consistent with previous findings [48] where concentrations of 2.5 mmol/L or higher inhibited end product formation in *Neocallimastix frontalis* RE1 on filter paper substrates. Notably, in this study, 3 mmol/L coumarin did not inhibit the growth of *P. ruminantium* F1. The use of readily degradable glucose as a carbon source may explain for some of the differences observed between our results and those of previous studies [48]. In anaerobic fungi, lactate and ethanol are primary products of cytosolic metabolism, whereas acetate is mainly produced in the hydrogenosomes [24]. The observed increase in lactate and ethanol and decrease in acetate suggests that coumarin primarily inhibits hydrogenosomal metabolism. The hydrogenosome is a critical metabolic organelle that produces ATP for the growth of anaerobic rumen fungi [49]. Thus, the coumarin tolerance of *P*. *ruminantium* F1 is closely related to its hydrogenosome metabolism. A previous study reported that high coumarin concentrations inhibited intracellular malate oxidation in bacterial communities from cattle manure, leading to malate accumulation [50]. This type of metabolic change could be the reason for the depressed growth of *P*. *ruminantium* F1, since coumarin above 3 mmol/L promoted cytosolic metabolism of *P*. *ruminantium* F1 and inhibited its hydrogenosomes (Fig. 3G).

The tolerance of *P. ruminantium* F1 to 3 mmol/L coumarin allowed for an in-depth investigation of its specific transcriptomic changes under coumarin exposure. Most of the enrichments were derived from the pathways of genetic information processing and cellular processes in microbes such as ribosome assembly and biogenesis and cytoplasmic translation. The reason could be that coumarin is an antimicrobial agent, and its ring could inhibit the synthesis of microbial nucleic acids [51], although the results indicated that both gas production and cell weight of *P. ruminantium* F1 were not affected by the addition of 3 mmol/L coumarin (Fig. 3). In fact, previous studies reported that the carbohydrate metabolism of anaerobic fungi (*Neocallimastix* spp.) was inhibited by coumarin whether cellulose or glucose was used as the carbon substrate [48]. Similar results were also found in soil and plant experimental systems. High concentrations of coumarin (200 and 300 mg/kg) increased soil fungal richness and altered the carbon metabolism of soil microbes [52]. Based on the CAZyme database, the differential gene expression caused by the addition of 3 mmol/L coumarin mainly belonged to the GH11, GH43, CE6, CBM6, and GH3 families, resulting in the up-regulated genes of EC 3.1.1.72 (acetyl xylan esterase), EC 3.2.1.8 (endo-β-1,4-xylanase), EC 3.2.1.37 (β-xylosidase), and others (Fig. S6). These results indicated that coumarin induced the increased fiber-degrading ability, as many of the upregulated genes are critical for hemicellulose degradation [18].

Previous studies have reported the metabolic pathways of coumarin by the enzymes secreted by microbes, especially *Pseudomonas* isolated from soil [12, 14]. In the first pathway, coumarin is converted to dihydrocoumarin by NAD(P)-dependent oxidoreductase (EC 1.1.1.-) secreted by microbes such as *Pseudomonas* sp. USTB‑Z or *Saccharomyces cerevisiae* strain [14, 34]. Subsequently, dihydrocoumarin is converted to melilotic acid under the catalysis of dihydrocoumarin hydrolase (EC 3.1.1.35) [13, 34]. The other metabolic pathway of coumarin is that coumarin is converted to *o*-coumaric acid and subsequently to melilotic acid, as observed in the metabolism of *Arthrobacter* spp. [53]. In this study, the relative concentration of *o*-coumaric acid increased until d 3, while melilotic acid increased after the degradation of *o*-coumaric acid (d 5). The above results indicated that the metabolic pathway of the anaerobic fungus *P. ruminantium* F1 was the same as that of *Arthrobacter* spp. [53], but different from that of *Pseudomonas* spp. (Fig. 5C) [34]. A previous study reported that dihydrocoumarin was hydrolyzed to melilotic acid by dihydrocoumarin hydrolase (EC 3.1.1.35) [54]. Zhao et al. [34] reported that the alpha/beta hydrolases of *Pseudomonas* sp. USTB‐Z opened the ring of dihydrocoumarin to produce melilotic acid during coumarin biodegradation. In the present study, *P. ruminantium* F1 had 8 genes encoding for the alpha/beta hydrolase family [24], among which 2 genes were significantly upregulated by the addition of coumarin (log_2_FC > 2.5, FDR < 0.01; Fig. 5B). This result suggested that these 2 genes may be crucial for catalyzing the conversion of coumarin to *o*-coumaric acid. Figure 5B also showed 5 upregulated genes encoding NAD(P)H oxidoreductase, which had the potential to convert *o*-coumaric acid to melilotic acid. In conclusion, this study showed that *P. ruminantium* F1 could convert coumarin to *o*-coumaric acid by the alpha/beta hydrolases, and subsequently to melilotic acid using the NAD(P)H oxidoreductases (Fig. 5C). It is also noteworthy that *P. ruminantium* F1 could not utilize coumarin as a primary energy substrate. The reason could be that the anaerobic fungus *P. ruminantium* F1 could not gradually convert melilotic acid to acetyl-CoA with the product pyruvate like *Pseudomonas* spp. [12], because the hydroxylation of melilotic acid is aerobic during this process [34].

## Conclusion

This study highlights the ability of rumen anaerobic fungi, specifically *Pecoramyces ruminantium* F1, to degrade coumarin, a phytotoxic secondary metabolite in *Melilotus officinalis*. The research showed that coumarin could not serve as a primary energy source for the fungus. However, despite the inhibitory effects of high concentrations, *P. ruminantium* F1 showed tolerance to 3 mmol/L and efficiently degraded coumarin through metabolic pathways involving alpha/beta hydrolases and NAD(P)H oxidoreductases. The fungi were able to metabolize coumarin to less toxic compounds, such as *o*-coumaric acid and melilotic acid. These findings challenge the traditional view of anaerobic fungi, which has primarily emphasized their lignocellulose-degrading abilities, while overlooking their detoxification potential. Future research will aim to unravel the specific molecular mechanisms underlying coumarin tolerance and degradation in rumen fungi.

## Supplementary Information

Supplementary Material 1: Fig. S1. Comparisons of gas production and metabolite concentrations from in vitro incubation of enriched rumen fungi and whole rumen microbiota with *Melilotus officinalis* as the substrate. Fig. S2. Dynamic changes of nutrients during *Melilotus officinalis* silage fermentation for 60 d. Fig. S3. Dynamic changes of nutrient retention rate and coumarin degradation rate of *Melilotus officinalis* silage fermented for 60 d. Fig. S4. Dynamic changes of pH value, lactateand NH3-Nconcentrations in *Melilotus officinalis* silage fermented for 60 d. Fig. S5. Effects of coumarinand glucoseon the biofilm morphology and metabolites of *P. ruminantium* F1. Fig. S6. Effects of coumarinon the transcripts and metabolic pathways of *P. ruminantium* F1. Fig. S7. Pathway enrichment analysis of genes from *P. ruminantium* F1 caused by the addition of 3 mmol/L coumarin at mid-exponential phase of fungal growth.

## Data Availability

The datasets generated and analysed during the current study are available in the NCBI Sequence Read Archive (SRA) database (Accession Number: PRJNA1033184), https://www.ncbi.nlm.nih.gov/bioproject/PRJNA1033184.

## Abbreviations

ADF: Acid detergent fiber
CAZymes: Carbohydrate-active enzymes
CP: Crude protein
DM: Dry matter
DMSO: Dimethylsulfoxide
NDF: Neutral detergent fiber
UPLC-MS: Ultra-performance liquid chromatography-mass spectrum
VFA: Volatile fatty acid
WSC: Water-soluble carbohydrate
