# Supplementary material for: Detoxification of coumarins by rumen anaerobic fungi: insights into microbial degradation pathways and agricultural applications
Source: J Anim Sci Biotechnol. 2025 Apr 17;16:59. doi: 10.1186/s40104-025-01195-9 (PMC12004625; doi:10.1186/s40104-025-01195-9)
Supplement: Supplementary file 1 — Supplementary Material 1: Fig. S1. Comparisons of gas production and metabolite concentrations from in vitro incubation of enriched rumen fungi and whole rumen microbiota with Melilotus officinalis as the substrate. Fig. S2. Dynamic changes of nutrients during Melilotus officinalis silage fermentation for 60 d. Fig. S3. Dynamic changes of nutrient retention rate and coumarin degradation rate of Melilotus officinalis silage fermented for 60 d. Fig. S4. Dynamic changes of pH value, lactateand NH3-Nconcentrations in Melilotus officinalis silage fermented for 60 d. Fig. S5. Effects of coumarinand glucoseon the biofilm morphology and metabolites of P. ruminantium F1. Fig. S6. Effects of coumarinon the transcripts and metabolic pathways of P. ruminantium F1. Fig. S7. Pathway enrichment analysis of genes from P. ruminantium F1 caused by the addition of 3 mmol/L coumarin at mid-exponential phase of fungal growth. [file 40104_2025_1195_MOESM1_ESM.docx]

**Fig. S1** Comparisons of gas production and metabolite concentrations from in vitro incubation of enriched rumen fungi and whole rumen microbiota with *Melilotus officinalis* as the substrate

**Fig. S2** Dynamic changes of nutrients during *Melilotus officinalis* silage fermentation for 60 d. DM, dry matter; CP, crude protein; WSC, water soluble carbohydrates; NDF, neutral detergent fiber; ADF, acid detergent fiber. Red line, *Melilotus officinalis* with anaerobic fungus supernatant silage group; Blue line, control group

**Fig. S3** Dynamic changes in nutrient concentrations and coumarin degradation in *Melilotus officinalis* silage fermented for 60 d. Red line, *Melilotus officinalis* with anaerobic fungus supernatant silage group; Blue line, control group

**Fig. S4** Dynamic changes of (**A**) pH value, (**B**) lactate and (**C**) NH_3_-N concentrations in *Melilotus officinalis* silage fermented for 60 d. Red line, *Melilotus officinalis* with anaerobic fungus supernatant silage group; Blue line, control group


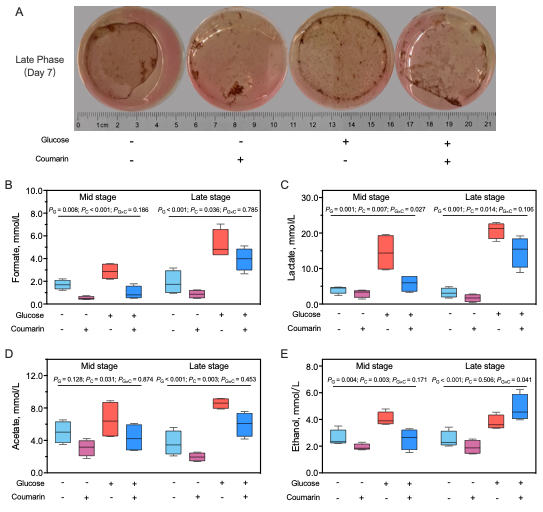


**Fig. S5** Effects of coumarin (5 mmol/L) and glucose (55 mmol/L) on the biofilm morphology and metabolites of *P*. *ruminantium* F1

**
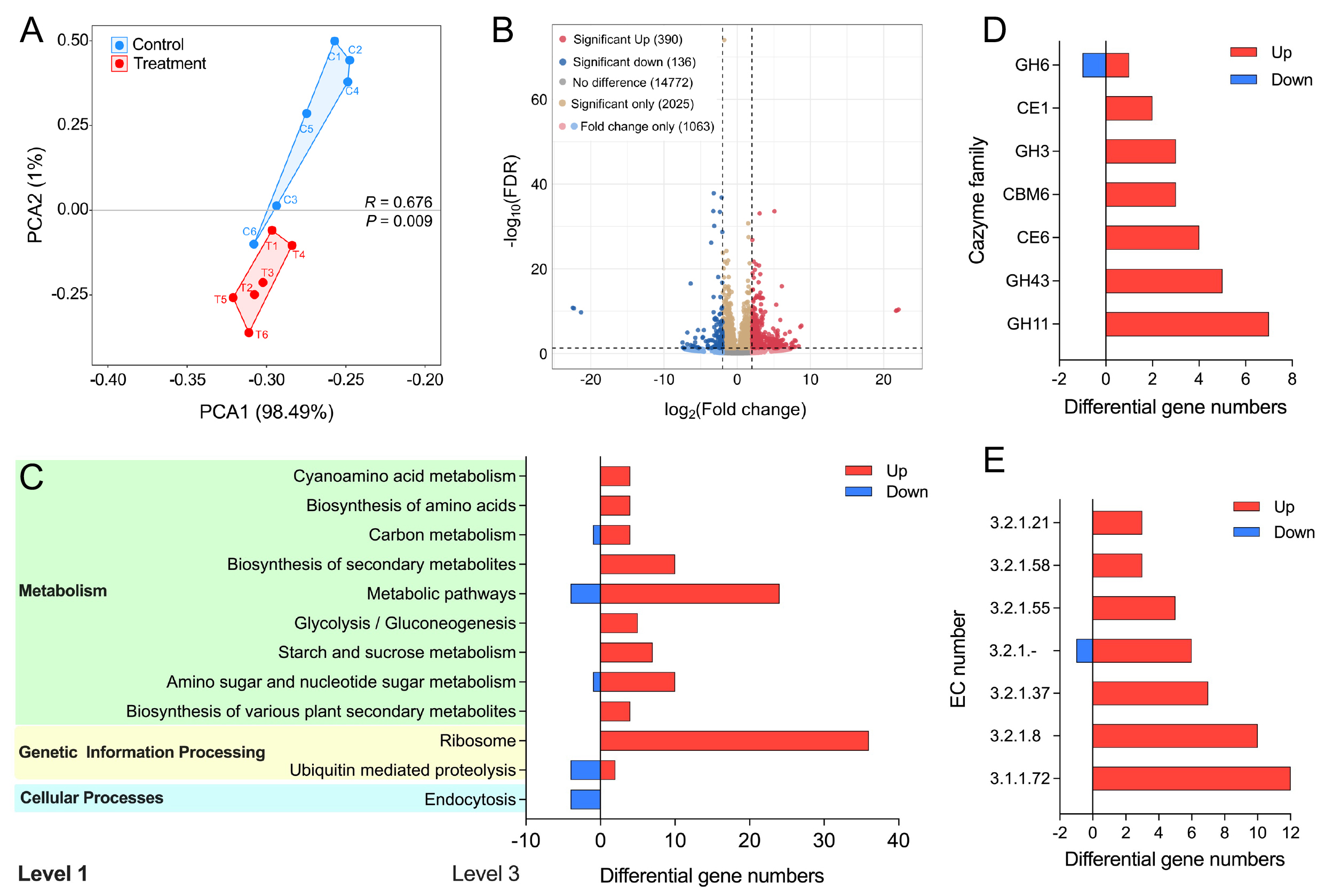
**

**Fig. S6** Effects of coumarin (3 mmol/L) on the transcripts and metabolic pathways of *P*. *ruminantium* F1. **A** Principal component analysis (PCA) of fungal genes. **B** Volcano plot for differential genes with FDR < 0.05 and | log_2_(fold change) | > 2. Differential genes classified into (**C**) KEGG level-3 pathways, and (**D**, **E**) Major carbohydrate-active enzymes


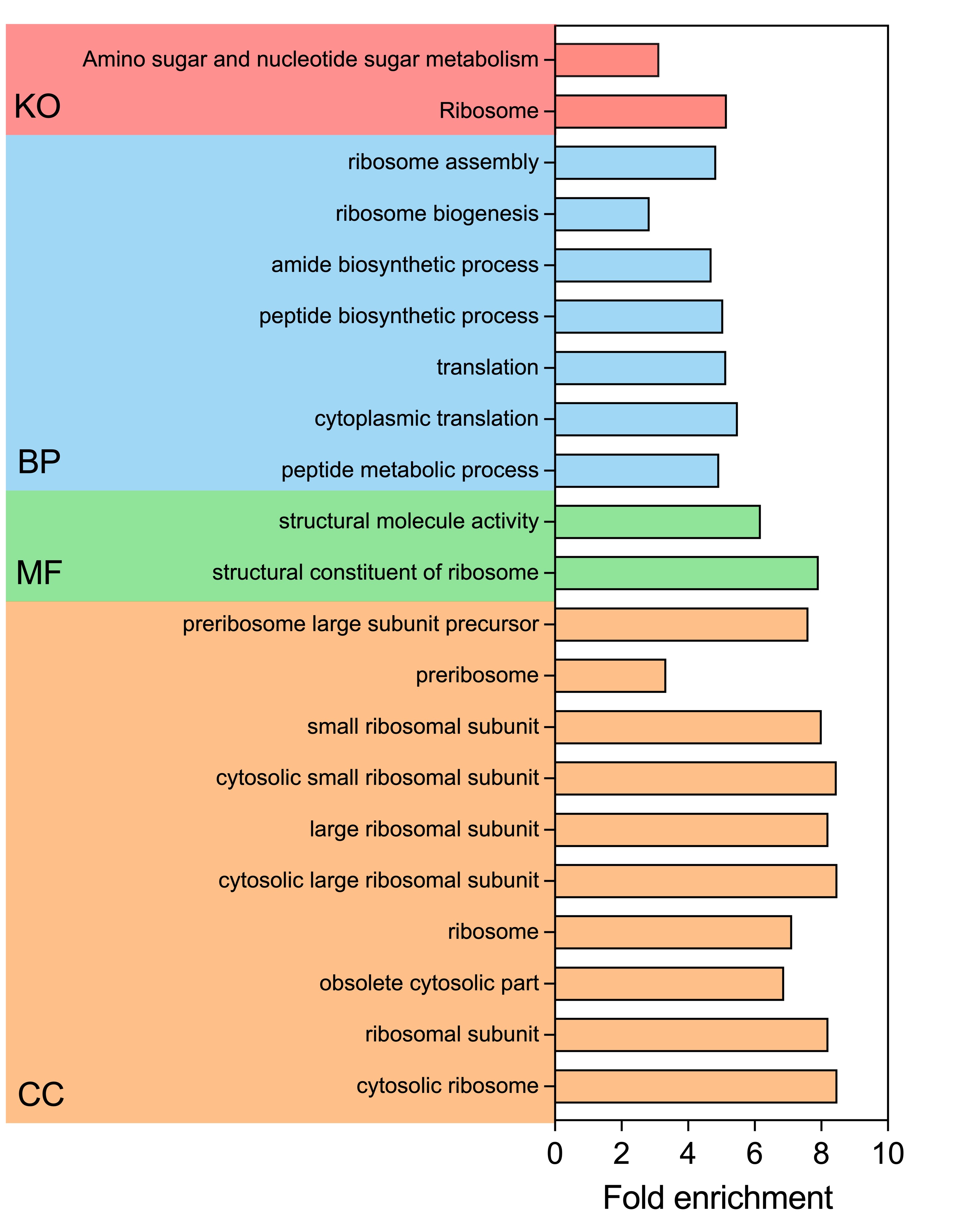


**Fig. S7** Pathway enrichment analysis of genes from *P*. *ruminantium* F1 caused by the addition of 3 mmol/L coumarin at mid-exponential stage of fungal growth. Only pathways with adjusted *P* values < 0.05 and fold enrichment > 2 were showed in this figure. KO, KEGG Orthologs; BP, biological process; MF, molecular function; CC, cellular component
